# Radioligands Targeting Fibroblast Activation Protein (FAP)

**DOI:** 10.3390/cancers13225744

**Published:** 2021-11-16

**Authors:** Thomas Lindner, Frederik L. Giesel, Clemens Kratochwil, Sebastian E. Serfling

**Affiliations:** 1 Department of Nuclear Medicine, University Hospital Würzburg, 97080 Würzburg, Germany; serfling_s1@ukw.de; 2 Department of Nuclear Medicine, University Hospital Düsseldorf, 40225 Düsseldorf, Germany; frederik.giesel@med.uni-duesseldorf.de; 3 Department of Nuclear Medicine, University Hospital Heidelberg, 69120 Heidelberg, Germany; clemens.kratochwil@med.uni-heidelberg.de

**Keywords:** FAP, cancer associated fibroblasts, radiopharmaceuticals, drug discovery

## Abstract

**Simple Summary:**

FAP-targeted radiotracers, recently introduced in cancer treatment, accumulate in Cancer-Associated Fibroblasts (CAFs). CAFs are present in tumor lesions but do not correspond to genuine cancer cells, although they behave in an abnormal and disease-promoting manner. One of their characteristic features, the expression of the surface protein FAP, can be utilized to discriminate between cancerous and healthy tissues. By the choice of an appropriate radionuclide, FAP-targeted tracers can be used for imaging or therapy in many cancer types. Therefore, the first successful application of FAP-targeted imaging has led to an enormous and growing interest in nuclear medicine and radiopharmacy.

**Abstract:**

Targeting fibroblast activation protein (FAP) in cancer-associated fibroblasts (CAFs) has attracted significant attention in nuclear medicine. Since these cells are present in most cancerous tissues and FAP is rarely expressed in healthy tissues, anti-FAP tracers have a potential as pan-tumor agents. Compared to the standard tumor tracer [^18^F]FDG, these tracers show better tumor-to-background ratios (TBR) in many indications. Unlike [^18^F]FDG, FAP-targeted tracers do not require exhausting preparations, such as dietary restrictions on the part of the patient, and offer the possibility of radioligand therapy (RLT) in a theragnostic approach. Although a radiolabeled antibody was clinically investigated as early as the 1990s, the breakthrough event for FAP-targeting in nuclear medicine was the introduction and clinical application of the so-called FAPI-tracers in 2018. From then, the development and application of FAP-targeted tracers became hot topics for the radiopharmaceutical and nuclear medicine community, and attracted the interest of pharmaceutical companies. The aim of this review is to provide a comprehensive overview of the development of FAP-targeted radiopharmaceuticals and their application in nuclear medicine.

## 1. Introduction

Fibroblasts are present in almost all tissues and usually rest in a quiescent stage. Following an insult of tissue integrity, fibroblasts become activated, migrate towards the site of the injury and orchestrate the repair of the damage. After the repair, the fibroblasts go back to their quiescent state, while in the case of chronic inflammation or fibrosis, they remain in an activated state. At the onset, cancer cells actively recruit fibroblasts by the secretion of chemokines or growth factors, such as fibroblast growth factor or transforming growth factor β. In the course of the disease, these cancer-associated fibroblasts (CAFs) start to recruit other fibroblasts and become involved in many other functions, which are responsible for the malignancy of a tumor. Their contributions to immune evasion and chemo-resistance, as well as tissue remodeling, are important drivers for invasiveness and metastasis [1]. One key instrument for the pro-tumorigenic role of CAFs is the fibroblast activation protein (FAP), which is a membrane-bound protease that cleaves peptide hormones, such as neuropeptide Y or substance P. FAP further degrades collagens after their denaturation by matrix metalloproteinases and, additionally, contributes to tumor growth in a non-enzymatic fashion [2]. While its biological role was initially unknown, it was later identified as a member of the dipeptidyl peptidase 4 (DPP4)-family [3,4]. Its expression pattern and distinct endopeptidase function make FAP a promising biomarker and target for many medical interventions [5].

**Figure 1:**
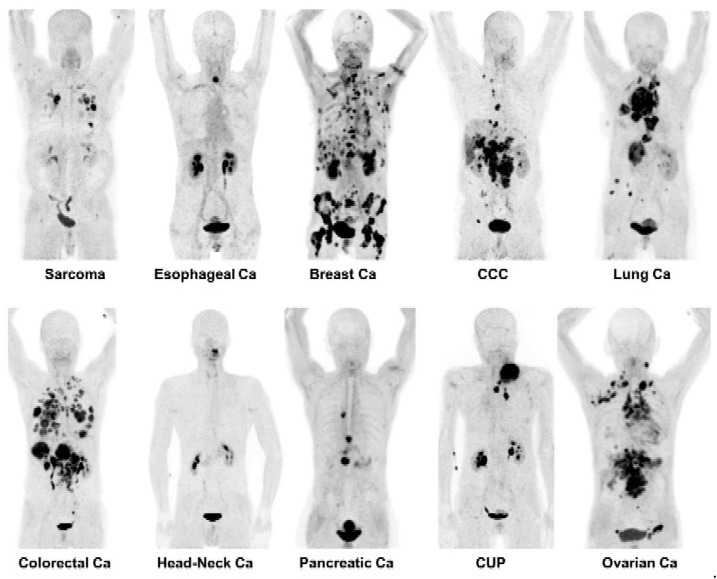
PET imaging with ^68^Ga-labeled FAPI-tracers. By targeting the cancer-associated fibroblasts while providing a low uptake in non-target organs and a fast background clearance, the FAPI-tracers allowed the detection of different tumor entities with a high specifity. Picture sections were taken from Kratochwil et al.; copyright Society of Nuclear Medicine & Molecular Imaging [6].

As shown in Figure 1, which is an excerpt of the study “^68^Ga-FAPI PET/CT: Tracer Uptake in 28 Different Kinds of Cancers” by Kratochwil et al., FAP-targeting possesses a pan-tumor applicability. Furthermore, this study showed that FAP-targeting is potentially superior to [^18^F]2-fluoro-2-deoxyglucose ([^18^F]FDG) in PET imaging for many important tumor entities, such as breast, lung, head-and-neck or colorectal cancers [6]. Although the number of publications is still growing, the small and heterogeneous patient collectives is still limiting the overall picture. Nevertheless, the potential for detection of primary and metastatic lesions is remarkable. However, for nodal detection and in general, more extended and indication-related prospective clinical trials are necessary [7].

Besides iodine-131, which is used in the therapy of thyroid cancers, radioligand therapies, like the ^153^Sm complex Quadramet or the ^90^Y-labeled antibody Zevalin, played a minor role as orphan drugs for a long time. Recently, the approval of Lutathera and the phase III VISION trial of ^177^Lu-PSMA-617 turbocharged commercial interest in nuclear medicine. Due to the above-discussed promises of FAP-targeting, SOFIE licensed the FAPI-compounds from Heidelberg and Clovis Oncology and the tracer FAP-2286 from 3B Pharmaceuticals, both at an early stage of development [8]. In the last months, Novartis sub-licensed the therapeutic application of the FAPI-tracers, and further FAP-radiotracers with alternative structures entered the market. This review will provide an overview of state-of-the-art radiotracer development in the literature and some highlights of clinical applications.

## 2. FAP Targeting Compounds

### 2.1. Antibodies

The first account of clinical FAP-targeting, together with planar and SPECT imaging in 17 patients before surgery, was published in 1994. Welt et al. reported that the ^131^I-labeled monoclonal murine antibody mAb F19 was able to detect lesions of a diameter as small as 1 cm at 3–5 days after injection [9]. Due to the encouraging results, sibrotuzumab, a humanized version of mAb F19, was developed to address the issue of immune response to murine antibodies. A phase I clinical study used co-administered ^131^I-labeled sibrotuzumab to evaluate pharmacokinetics. Since only two out of 26 patients showed temporary stable disease, the end point of the study was not reached [10].

Recently, Pandya et al. updated the mAb F19 antibody with a desferrioxamine (DFO) chelator for the complexation of the positron emitter ^89^Zr, which has a half-life suitable for PET imaging with radiolabeled antibodies [11].

Hintz et al. validated metastatic castration-resistant prostate cancer (mCRPC) as a target for anti-FAP theragnostics by means of immunohistochemistry and qRT-PCR. Hereafter, a ^89^Zr-labeled antibody, which was originally identified by phage display, was used for PET/CT imaging in prostate cancer xenograft models. The best time point for imaging was at 3 days after injection, while tumor-to-background values decreased for some tissues afterwards. The highest non-target accumulation was detected in the liver. Nevertheless, the presented clearance from blood is fast compared to ^89^Zr-labeled mAb F19. This lowers the dose absorbed by radiation sensitive bone marrow, while, additionally, the application of conjugates with cytotoxic drugs were recommended [12].

### 2.2. Peptides

Although radiolabeled antibodies and their fragments offer high target affinity and selectivity, the long circulation and slow clearance caused by the high molecular mass are hampering the wider application in nuclear medicine. Additionally, the use of nuclides with longer half-lives, e.g., ^89^Zr or ^227^Th, are challenging for waste and spill management. Peptides are an alternative to overcome these drawbacks. Biotechnological as well as chemical approaches and their combinations are applicable for the identification and development of suitable peptide-based tracers.

For example, in 2020 3B-Pharmaceuticals GmbH (Berlin, Germany) described a series of peptides possessing a cyclic heptapeptide core with nanomolar IC_50_ values against FAP. The cyclisation is achieved by thioetherification of N- and C-terminal cysteine residues with derivatives of 1,3-bis-(bromomethyl)-benzene. The enclosed five amino acids were optimized for affinity, and further residues were added at the outside of the ring to enhance pharmacokinetic properties. Since the patent uses a different nomenclature, the clinical candidate among the substances, FAP-2286, cannot be identified with absolute certainty [13]. The slow tumor-washout of the lead compound, as well as the successful radioligand therapy in patient derived xenografts, were also the subject of a poster presentation at the ESMO 2020 virtual congress [14].

### 2.3. Small Molecules

**Figure 2:**
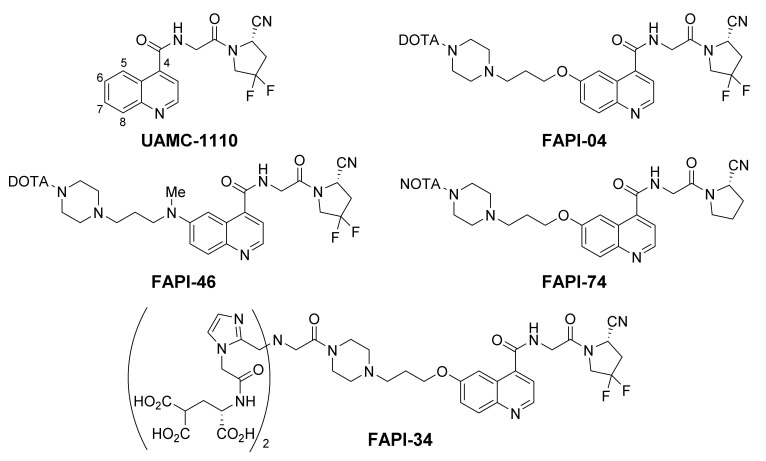
UAMC-1110, developed at the University of Antwerp, and various FAPI-tracers, developed by the Haberkorn group, based on this motif. FAPI-04 was the first theragnostic anti-FAP agent and is currently the most investigated tracer. FAPI-46 offers improved tumor retention and pharmacokinetics; FAPI-74 can be labeled with [^18^F]aluminum fluoride or ^68^Ga at ambient temperature; FAPI-34 is suitable for labeling with the theragnostic pair ^99m^Tc/^186/188^Re.

Small molecule drugs further reduce the molecular weight, which improves tissue penetration and eventually pharmacokinetics of the derived radiotracers. With FAP belonging to the family of serine proteases, several inhibitors against its enzymatic function have been described in the literature. The highly selective and potent inhibitors based on a (4-quinoinolyl)glycinyl-2-cyanopyrrolidine scaffold, developed by the group of van der Veken, are of special interest [15,16]. As shown in Figure 2, the FAPI-precursors were designed on the basis of this motif. The name “FAPI” is an acronym for “FAP inhibitor”, which was chosen by the Heidelberg group and was also adapted by other groups for the description of radiotracers, which are 6-quinolyl modified derivatives of UAMC-1110.

Due to the previously published results, quinoline position 6 and 7 were chosen by the Heidelberg group to attach the chelator moiety with intention of optimizing the linker structure [17]. The best results were obtained with a 1,3-propylen spacer between a *N*-piperazine and an ether oxygen or a methylamino group bound to the quinoline. For example, shorter spacers drastically reduced in vitro uptake, a 4-piperidine instead of the piperazine led to higher background in xenotransplants and the 2,6-methylene bridged piperazine of FAPI-21 led to a higher accumulation in salivary glands [17,18].

Currently, FAPI-04 is the most clinically investigated tracer, with a still growing number of publications. It was the first small molecule tracer against FAP that was also used in a therapeutic setup [17]. Additionally, experiments in a pancreatic cancer xenograft model using ^64^Cu and ^225^Ac for imaging and a proof-of-concept therapeutic study have been reported [19]. Initially, ^68^Ga-FAPI-02 was successfully used in small animal PET imaging, as well as in clinical PET/CT, but a fast washout from tumor tissues prevented therapeutic applications [20]. By means of the difluoro substitution at the cyanopyrrolidine moiety, a reduction of the IC_50_ value occurred, which was comparable to the non-radioactive analogue reported by Jansen et al. [16,17]. Fortunately, this effect was accompanied by a major improvement in tumor retention of the radioligand but without an interference with background clearance. FAPI-46 was developed to further extend tumor retention while maintaining the diagnostic benefit of a fast clearance. This was achieved by a methylated nitrogen, which replaced the bridging oxygen at the 6-quinoline position. By slight changes of the physicochemical properties, the Haberkorn group expanded the structure-activity relationship of the linker region and identified a compound with improved pharmacokinetics [18].

Since ^18^F-tracers offer benefits like larger batch size, longer half-life and lower positron energy than ^68^Ga, the implementation of ^18^F-labeled FAP-tracers is also a subject of research. Toms et al. used a copper catalyzed cycloaddition to obtain the radiotracer [^18^F]FGlc-FAPI. In this reaction, 6-deoxy-6-[^18^F]fluoroglycosyl azide and an alkyne precursor similar to FAPI-04 form a triazole linker connecting each other. While in vitro experiments showed promising results, a pronounced hepatobiliary excretion together with low tumor uptake were observed for [^18^F]FGlc-FAPI in xenograft studies [21].

In order to minimize the change of the pharmacophore, the chelation of an aluminum fluoride complex offers an attractive alternative to a covalent fluorination. Due to the incompatibility of the DOTA chelator with aluminum fluoride labeling, a different chelating group has to be attached, one of which is the smaller homologue NOTA. While [^18^F]AlF-FAPI-42, the NOTA-derivative of FAPI-04, showed hepatobiliary excretion, Lindner et al. succeeded with FAPI-74, the analog precursor lacking the difluoro substitution at the pyrrolidine ring. For the corresponding radiotracers [^18^F]AlF-FAPI-74 and ^68^Ga-FAPI-74, Giesel et al. demonstrated an equal performance compared to ^68^Ga-FAPI-04 PET/CT in clinical applications [22,23]. Independently, Jiang et al. reported preclinical results and a first-in-man study of [^18^F]AlF-labeled NOTA-FAPI-04 (FAPI-42) [24].

Further investigations included the incorporation of other common radionuclides. While PET/CT scanners are expensive and fewer in number, SPECT/CT scanners together with the ^99^Mo/^99m^Tc-generators are more broadly available and represent the backbone of nuclear medicine. To label with ^99m^Tc and potentially enable stable complexation of ^186/188^Re in the future, Lindner et al. designed various precursors carrying bisimidazole chelators. The most successful candidate was FAPI-34, which compensated the high lipophilicity of the ^99m^Tc-tricarbonyl core with two carboxyglutamate residues attached to the chelating moiety [25].

**Figure 3:**
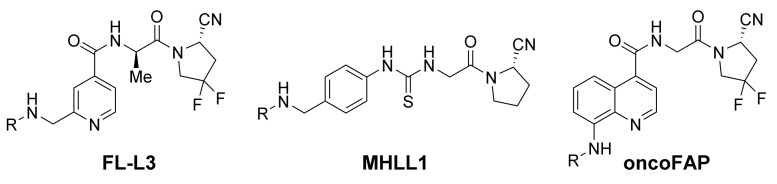
Small-molecule FAP-targeted radiotracers published by Roy, Langer and Millul [26,27,28]. R represents the chelating moiety, which is eventually attached by an appropriate linker.

The group of Philip S. Low published an alternative ^99m^Tc-tracer. The FAP-tracer FL-L3 (Figure 3) forms a complex with ^99m^Tc by a tripeptide moiety (2,3-diaminopropanoic acid-aspartate-cysteine), which is attached by a glutaric acid/8-aminooctanoic acid linker. Additionally, this tracer uses a (2-aminomethylene-4-pyridinolyl)-d-alanyl-2-cyanopyrrolidine instead of the UAMC1110-scaffold. In comparison, kidney uptake was relatively high in a biodistribution study in MDA-MB-231 xenografts. Due to a lack of further time points, no trend regarding tissue retention of this radiotracer is evident. Nevertheless, a derivative coupled to the cytotoxic drug tubulysin was effective in a therapeutic study with the MDA-MB-231 xenograft [26].

Another precursor lacking the quinoline motif is MHLL1 (Figure 3). Langer et al. used the ^68^Ga-labeled tracer for FAP-targeted monitoring of fibroblast activity after myocardial infarction. In this case, the quinolinoyl moiety was replaced by (4-aminomethylphenyl)thiourea, which is introduced by the commercially available p-SCN-Bn-NODAGA building block. Although the synthesis reduces the total number of steps, problems regarding the purification of the final precursor by means of preparative HPLC were reported. While the compound showed a partial biliary elimination and was not tested in an oncological setup, the PET/CT-imaging of the infarcted region at 7 and 21 days post infarction were successful [27].

In the case of the tracer oncoFAP (Figure 3), introduced by PhiloChem, an amino group in the 8-quinoline position served as the attachment point for fluorescent labels, chelator moieties or the cytotoxic drug vedotin. Although Jansen et al. reported a loss of potency and selectivity for halogen substituents at the 8-position [15], high tumor-to-background ratios were observed in imaging and biodistribution experiments in xenografts with transfected cell lines. The publication also shows therapeutic efficiency of a small-molecule–vedotin conjugate in combination with an antibody–cytokine fusion protein. While the company announced first-in-man imaging studies in Munster (Germany), only preclinical data are available in the literature up to now (end of July 2021) [28].

**Figure 4:**
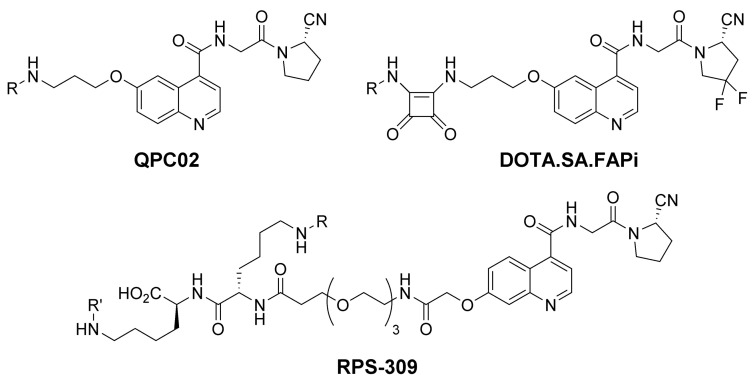
FAP-targeted precursors using a 6- or 7-alkoxy substituted quinoline backbone. R represents the individual chelating moiety. In the case of RPS-309, R′ represents an albumin binding moiety.

The Pomper group developed the DOTAGA armed precursor QCP02 (Figure 4) together with a near-infrared probe QPC01. As in the case of the FAPI-precursors, both use the 6-quinoline position for the linker attachment but lack the piperazine ring. For radiolabeling, ^111^In was used for preclinical experiments after the removal of an unlabeled precursor by means of HPLC. While PC-3 FAP negative tumors showed low uptake, the uptake in the co-transplanted FAP positive U87MG xenotransplants was significantly higher. Thus, a considerable wash out and hepatobiliary excretion were noticed in small animal SPECT/CT and biodistribution studies [29].

The radiotracers reported by Moon et al. use a squaric acid bisamide instead of the piperazine moiety of FAPI-04. In addition to DOTA, a modified version of the DATA chelator was also introduced to enable ^68^Ga-labeling at room temperature. Since only one time point for imaging and biodistribution experiments of DOTA.SA.FAPi (Figure 4) was reported, a chronological trend of the uptake cannot be deduced. Furthermore, an uptake in the small intestine, as well as off-target accumulations in the normalized coronal 2D-PET/CT images found in the supporting information, remained unexplained [30].

To increase the dose delivered to the tumor, Kelly et al. developed RPS-309 (Figure 4), which additionally binds to albumin in order to slow clearance. A small PEG-unit at the 7-quinoline position was used to attach two sidechain-modified lysine residue. One lysine carrying the chelator, the other acylated with (4-iodophenyl)acetic acid. The albumin binding capacity of this moiety slows clearance to potentially deliver higher doses to the tumors in therapeutic applications. While this strategy was successful for other radiotracers, fast tumor washout of RPS-309 combined with higher non-target exposure to prohibit its use in theragnostics [31].

It is difficult to decide which radiotracer is advantageous since most working groups tested their individual tracers in different preclinical setups. Factors that affect the results are the expression rate of the FAP positive cells, choice of nuclide or specific activity, all of which lead to different results. Another factor is the statistical uncertainty, due to the small number of animals used in each xenograft experiment. In addition, the time span between the tumor implantation and the experiment can be a decisive factor too. Ding et al. noticed that the ideal time for FAPI imaging spanned approximately a week in a longitudinal imaging study performed in a syngeneic mouse model for metastatic breast cancer [32]. With the anticipation of these uncertainties, the development of the FAPI-tracers focused more on tumor retention and pharmacokinetics than the absolute uptake or IC_50_ values. Another aim was to exclude hepatobiliary excretion and, especially, to reduce the exposure of non-target tissues, such as bone marrow or salivary glands [17,18,23,25].

## 3. Clinical Applications of Peptide and Small-Molecule Tracers

### 3.1. Benefit of FAP-targeting in Clinical Imaging

The number of FAPI publications is growing rapidly. A PubMed search for the term ‘FAPI PET’ listed three publications for 2018, 13 in 2019, 51 in 2020 and 122 (already) at the end of July 2021. The largest fraction of the publications deals with the clinical application of the ^68^Ga-labeled tracers FAPI-04 and FAPI-46, of which single case reports correspond to the majority of publications. In the following, a set of publications is highlighted to provide an overview of the benefits and applicability that FAP-targeting offers to nuclear medicine. Possible pitfalls in FAP-targeted diagnostics are comorbidities, which are accompanied by tissue rearrangements, such as fibrosis, some forms of rheumatism or chronic pancreatitis. Although this fact has to be carefully taken into account for tumor staging and other purposes, the involvement in many non-malignant conditions is also of interest beyond the field of oncology [33].

Although the Standardized Uptake Values (SUVs) are similar between FAP-targeted tracers and [^18^F]-FDG, the tumor-to-background ratios are considerably higher due to a lower accumulation in non-target tissues [34]. Imaging offers the best results in a time window of approximately 30–180 min after tracer injection, which is a significant advantage in terms of scheduling in clinical practice. Furthermore, the practical disadvantage of FDG, the requirement of a low blood sugar level is overcome. Fasting for at least six hours with around two hours for distribution and image acquisition, can be challenging particularly for diabetic patients who have cancers. While being indifferent to the metabolic activity of healthy tissues or inflammation, FAP-targeting focuses on tissue remodeling activity. These facts make these tracers appear as tailor-made for the detection, stratification and therapy follow-up examinations.

Due to its high glucose metabolism, the brain parenchyma has a high avidity for [^18^F]FDG. In the case of lung cancer, which has a high tendency for spreading to the brain, an additional contrast-enhanced MRI should be performed, to rule out undetected cerebral metastasis. As shown by Giesel et al., FAP-targeted PET/CT clearly visualized two lesions, one as small as 8 mm in diameter, both of which were not delineable in non-enhanced CT [35].

A further case of physiological [^18^F]FDG avidity is inflammation. Malignancies that occur with a neighboring inflammation can lead to imprecise tumor delineation in [^18^F]FDG imaging. Examples are head-and-neck cancers where FAP-targeted tracers could help to distinguish exactly the tumorous tissue preoperatively/before radiation from inflammation tissue, which leads to an altered target [36].

**Figure 5:**
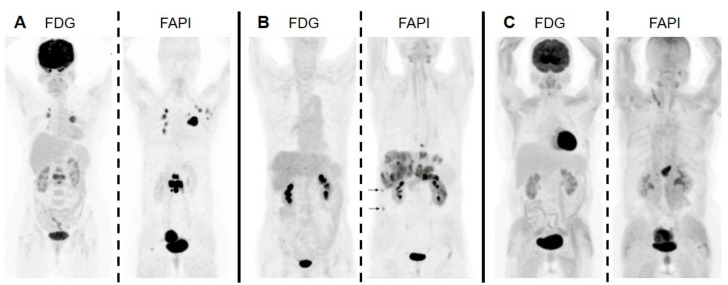
Cases in which FAP-targeted imaging outperformed [^18^F]FDG PET/CT. (**A**) Invasive lymphnodal metastatic duct breast carcinoma. (**B**) Pancreatic duct adenocarcinoma with liver metastases. (**C**) Poorly differentiated gastric adenocarcinoma. Image sections were taken from Kömek et al., Chen et al. and Kuten et al.; copyright Springer-Verlag GmbH Germany, part of Springer Nature [38,39,40].

As the delineation of the tumor area in imaging with [^18^F]FDG can be imprecise due to background avidity, it can be impaired likewise by a reduced uptake in tumor tissues. For example, remaining glucose-6-phosphatase activity prevents [^18^F]FDG from accumulation in tumor lesions like low-grade hepatocellular carcinomas [37]. Since FDG often shows a limited detection rate of the tumor lesions with a low Warburg effect, FAP-targeted imaging has the potential to be a complementary imaging modality in these cases. As in the instance of the pancreatic cancer patient in Figure 5, liver metastases became detectable, which were missed by [^18^F]FDG [38].

Due to heterogeneity in glucose consumption and uptake in benign pathologies, the imaging of gynecological cancers by [^18^F]FDG PET/CT can also lead to inconclusive results, especially for small lesions or nodal metastases. Kömek et al. performed a clinical study with 20 breast cancer patients which compared ^68^Ga-FAPI-04 with [^18^F]FDG. Besides the higher tumor-to-background-ratios for each respective location, the FAP-targeted imaging had a higher detection rate for primary tumors and metastases in lymph nodes, lungs, brain and bone, as well as the liver [40].

In a retrospective analysis of 167 female patients with different tumors, Dendl et al. noticed a physiological uptake in hormone sensitive tissues, such as the ovaries, breast and especially the endometrium, which was highest in premenopausal women. Primarily, the study analyzed a different set of 31 patients with different gynecological tumors. In the cases of four patients with cervical and two patients with endometrial cancer, the physiological uptake of the surrounding tissue did not impair the sensitivity for FAP-targeted PET/CT of these two tumor entities. Overall, for the five indications covered, FAP-guided imaging was beneficial compared to [^18^F]FDG. This conclusion was drawn on basis of the sensitivity for primary tumors and metastases, tumor-background-ratios, as well as mean SUV_max_, for all lesions besides those in the lung [41].

As an alternative to the FAPI-compounds, Ballal et al. published a comparison of ^68^Ga-DOTA.SA.FAPI versus [^18^F]FDG in 54 patients. The dosimetric data in most tissues were reported as comparable with the previously published data for ^68^Ga-FAPI-04 and ^68^Ga-FAPI-46, while being noticeably higher for the liver, spleen and especially the healthy pancreas. Additionally, a single therapeutic application of 3.2 GBq ^177^Lu-labeled tracer was tolerated well by a breast cancer patient and resulted in a reduction in pain symptoms and medication [42].

As mentioned earlier, the number of patients in each study is relatively small, and heterogeneous malignancies were included in many publications. Another bias arises from the fact that often the patients for the FAP-imaging were recruited due to previous inconclusive results in FDG PET/CT. Further studies with a limited setting of disease are warranted to evaluate the benefits of FAP-targeting versus standard [^18^F]FDG PET/CT for a certain indication [7]. Nevertheless, FAP-targeted PET/CT is already a complementary imaging modality for cases in which a precedent examination with [^18^F]FDG PET/CT is ambiguous. These include hepatocellular and cholangiocarcinomas, gastric, pancreatic or gynecological cancers, while, additionally, cancers of unknown primary may be detectable as well as a peritoneal spread [6,38].

### 3.2. Therapeutic Application

Currently, only proof-of concept studies have been published for FAP-targeted radioligand therapies in a few cases. In a first case, a breast cancer patient treated with ^90^Y-labeled FAPI-04 showed a reduction of pain symptoms in response [17]. Another patient with ovarian cancer, who received ^90^Y-FAPI-46 as last line therapy showed stable disease in the 2-month follow-up by FAP-targeted SPECT/CT [25]. Although ^90^Y has a better-suited half-life for FAP-targeted therapy, ^177^Lu offers the possibility to irradiate smaller lesions more efficiently. Kuyumcu et al. reported a low-dose dosimetry study in four patients with ^177^Lu-FAPI-04. While the dose delivered to dose-limiting tissue was low, the average dose delivered to cancerous tissue was also low on average, with 0.33–0.62 Gy/GBq at different lesion sites, while individual lesions showed doses up to 1.67 Gy/GBq [43]. Assadi et al. used ^177^Lu-FAPI-46 for radioligand therapy in 18 patients with an average of 3.7 GBq administered to each in two treatment cycles on average [44].

Baum et al. reported the clinical application of ^68^Ga- and ^177^Lu-FAP-2286. In a retrospective study with 11 patients without further treatment options, the authors noted a significant reduction in pain for three but also new symptoms for two patients, and an average dose of 3.0 Gy/GBq in bone metastases. Additionally, G3 hematological side effects were reported, although ^177^Lu-dosimetry data showed low red marrow exposure, while the doses delivered to the kidneys were comparable to ^177^Lu-PSMA-617. After 6–8 weeks after the first therapy cycle, two out of 11 patients showed stable disease, of which one progressed 8 weeks after the third treatment cycle [45].

An initially promising alternative was the use of ^153^Sm. A complex of this nuclide with ethylene diamine tetramethylene phosphonate is used as Quadramet^®^ for pain reduction in the case of bone metastases. ^153^Sm has a half-life of 46.3 h, a beta-energy of 0.8 MeV and a SPECT compatible gamma emission at 103 keV. The common production uses the neutron irradiation of enriched ^152^Sm. Therefore, the nuclide is not available carrier free. As reported by Kratochwil et al., a patient with soft tissue sarcoma showed stable disease for a duration of 8 months while treated with accumulated 20 GBq ^153^Sm-FAPI-46 and 8 GBq ^90^Y-FAPI-46. Regarding technical issues, such as varying specific activities of the nuclide and ^154^Eu impurities, other alternative nuclides were recommended for future research [46]. In another promising approach, ^90^Y-FAPI-46 was combined with pembrolizumab. Although novel metastases were detected, the lesions avid prior to therapy showed a reduction in size and intensity in the follow up ^68^Ga-FAPI-PET/CT together with an improved overall condition [47].

The current therapeutic approaches are scarce while still in evaluation and will eventually only be considered as last-line treatment for a limited number of patients. Nevertheless, FAP-targeting has the potential to improve therapeutic outcomes for many patients already. The better tumor-to-background ratios help delineate the cancerous tissue, while the higher detection rate and lower false-positive rates improve the initial staging or restaging of a cancer. All these factors are essential for the planning of external beam radiation therapies. As reported by Giesel et al., Koerber et al. and Syed et al., the gross tumor volumes for radiotherapy planning were frequently significantly larger in FAPI imaging compared to the standard imaging modalities of contrast-enhanced CT and/or MRI. Since the cancer associated fibroblasts are directly involved in the invasion of surrounding tissues and resistance to various therapies, a boosted radiation in areas with high FAP-activity might reduce the risk of recurrence from residual disease [34,48,49].

## 4. Conclusions

Small-molecule and peptide-based tracers are currently the most promising groups for FAP-targeted theragnostics. Unfortunately, preclinical reports have employed different experimental setups, and only small sets of patients with different pathologies and morbidities took part in dosimetry studies. However, a slight tendency is apparent: antibodies and peptides show longer tumor retention but higher non-target exposure—red bone marrow for antibodies and kidneys for peptides. To overcome this fact, FAPI and other cyanopyrrolidine-based tracers might benefit from the current development of promising therapeutic nuclides. For example, ^47^Sc, ^67^Cu or ^212^Pb offer better-suited half-lives than ^177^Lu and higher linear energy transfer than ^90^Y. Nevertheless, all anti-FAP radioligand therapies might also benefit from a synergistic combination with non-radioactive cancer therapeutics. Due to the high tumor-to-background ratios in PET/CT or SPECT/CT imaging, they offer an improvement for cancer therapy management.

## Data Availability

Not applicable.
